# Ground-based microgravity and proton radiation exposure alters leukocyte activity

**DOI:** 10.1093/jrr/rrt218

**Published:** 2014-03

**Authors:** Jenine K. Sanzari, Ana Romero-Weaver, Gabriel S. Krigsfeld, Gabrielle James, Liyong Lin, Eric S. Diffenderfer, Ann R. Kennedy

**Affiliations:** Department of Radiation Oncology, Perelman School of Medicine, University of Pennsylvania, 195 John Morgan Building, 3620 Hamilton Walk, Philadelphia, PA 19104-6072

## Abstract

Immune system adaptation during spaceflight is a concern in space medicine. Decreased circulating leukocytes observed during and after space flight infer suppressed immune responses and susceptibility to infection. The microgravity aspect of the space environment has been simulated on Earth to study adverse biological effects in astronauts. In this report, the hindlimb unloading (HU) model was employed to investigate the combined effects of solar particle event-like proton radiation and simulated microgravity on immune cell parameters, including lymphocyte subtype populations and activity. Lymphocytes are a type of white blood cell critical for adaptive immune responses and T lymphocytes are regulators of cell-mediated immunity, controlling the entire immune response. Mice were suspended prior to and after proton radiation exposure (0 or 2 Gy doses) and total leukocyte numbers and splenic lymphocyte functionality were evaluated on days 4 or 21 after combined HU and radiation exposure. Total white blood cell (WBC), lymphocyte, neutrophil and monocyte counts are reduced by ∼65, 70, 55 and 70%, respectively, compared with the non-treated control group 4 days after combined exposure. Splenic lymphocyte subpopulations are altered at both time points investigated. At 21 days post-exposure to combined HU and proton radiation, T-cell activation and proliferation were assessed in isolated lymphocytes. Cell surface expression of the Early Activation Marker, CD69, is decreased by 30% in the combined treatment group, compared with the non-treated control group and cell proliferation was suppressed by ∼50%, compared with the non-treated control group. These findings reveal that the combined stressors (HU and proton radiation exposure) induce decreased leukocyte numbers and function, contributing to immune system dysfunction in crew members.

This research was supported by the NIH Training Grant 2T32CAO9677 and the National Space Biomedical Research Institute (NSBRI) Center of Acute Radiation Research (CARR) grant. The NSBRI is funded through the National Aeronautics and Space Administration (NASA) Class Code (NCC) 9-58.

The authors declare that this work has been published: Sanzari JK, Romero-Weaver AL, James G, Krigsfeld G, Lin L, Diffenderfer ES, Kennedy AR. Leukocyte activity is altered in a ground based murine model of microgravity and proton radiation exposure. PLoS One. 2013;8(8):e71757. doi:10.1371/journal.pone.0071757.

